# *ZRSR2* overexpression is a frequent and early event in castration-resistant prostate cancer development

**DOI:** 10.1038/s41391-021-00322-7

**Published:** 2021-02-10

**Authors:** Haiqing He, Jun Hao, Xin Dong, Yu Wang, Hui Xue, Sifeng Qu, Stephen Yiu Chuen Choi, Xinpei Ci, Yong Wang, Rebecca Wu, Mingchen Shi, Xiaokun Zhao, Colin Collins, Dong Lin, Yuzhuo Wang

**Affiliations:** 1 Vancouver Prostate Centre, Department of Urologic Sciences, University of British Columbia, Vancouver, BC Canada; 2 Department of Urology, The Second Xiangya Hospital, Central South University, Changsha, China; 3 Department of Experimental Therapeutics, BC Cancer Research Centre, Vancouver, BC Canada; 4 Department of Urology, Qilu Hospital, Cheeloo College of Medicine, Shandong University, Jinan, Shandong China; 5 School of Medicine, Cheeloo College of Medicine, Shandong University, Jinan, Shandong China

**Keywords:** Prostate cancer, Prostate cancer

## Abstract

**Background:**

Androgen deprivation therapy (ADT) remains the leading systemic therapy for locally advanced and metastatic prostate cancers (PCa). While a majority of PCa patients initially respond to ADT, the durability of response is variable and most patients will eventually develop incurable castration-resistant prostate cancer (CRPC). Our research objective is to identify potential early driver genes responsible for CRPC development.

**Methods:**

We have developed a unique panel of hormone-naïve PCa (HNPC) patient-derived xenograft (PDX) models at the Living Tumor Laboratory. The PDXs provide a unique platform for driver gene discovery as they allow for the analysis of differentially expressed genes via transcriptomic profiling at various time points after mouse host castration. In the present study, we focused on genes with expression changes shortly after castration but before CRPC has fully developed. These are likely to be potential early drivers of CRPC development. Such genes were further validated for their clinical relevance using data from PCa patient databases. *ZRSR2* was identified as a top gene candidate and selected for further functional studies.

**Results:**

*ZRSR2* is significantly upregulated in our PDX models during the early phases of CRPC development after mouse host castration and remains consistently high in fully developed CRPC PDX models. Moreover, high *ZRSR2* expression is also observed in clinical CRPC samples. Importantly, elevated *ZRSR2* in PCa samples is correlated with poor patient treatment outcomes. *ZRSR2* knockdown reduced PCa cell proliferation and delayed cell cycle progression at least partially through inhibition of the Cyclin D1 (*CCND1*) pathway.

**Conclusion:**

Using our unique HNPC PDX models that develop into CRPC after host castration, we identified *ZRSR2* as a potential early driver of CRPC development.

## Introduction

Androgen deprivation therapy (ADT) remains the first-line treatment for patients with locally advanced and metastatic prostate cancer (PCa) [1]. While a majority of patients initially respond well to ADT, most will progress to castration-resistant prostate cancer (CRPC). Over the last decade, the discovery that most CRPCs are still dependent on androgen and androgen receptor (AR) signaling led to the development of several new androgen receptor pathway inhibitors (ARPIs) such as abiraterone, enzalutamide and apalutamide [2–4]. While these next-generation ARPIs can effectively reduce symptoms and prolong life, metastatic CRPC (mCRPC) remains incurable as resistance to these ARPIs frequently emerges. Furthermore, although multiple ARPIs are currently in clinical use, most of these therapies focus on late stages of the disease. Knowledge surrounding the early stages of CRPC development remain limited. As such, a better understanding of the mechanisms that underlie the early phases of CRPC development is needed to develop novel therapeutic approaches.

It is particularly important, yet extremely challenging, to study the early stages of CRPC development. This is largely due to a difficulty in acquiring adequate clinical samples and a lack of suitable patient-derived xenograft (PDX) models that can represent the biology of patient CRPC progression. At the Living Tumor Laboratory (www.livingtumorlab.com), we have previously established a large panel of PDX models from various clinical stages of PCa. In particular, we have a unique panel of PCa PDXs originating from hormonal-naïve PCa (HNPC) tissues [5]. Overall, these PDXs recapitulate the heterogeneity of the patients’ original tumors in a high-fidelity manner [5]. More excitingly, the HNPC PDXs are also able to closely mimic the original tumor’s biological behaviors and disease progression, including development into CRPC following mouse host castration. Thus, such HNPC-to-CRPC PDXs provide an excellent opportunity to model CRPC progression and analyze gene expression changes longitudinally during this process (i.e., at multiple time points pre-castration, post-castration, and when fully-relapsed). This makes the identification of potential early driver genes that critically contribute to CRPC development possible.

In this study, we mimic CRPC progression using seven HNPC PDX models (i.e., LTL310, 311, 313B, 313H, 418, 467, and 484). Following host mouse castration, these HNPC PDXs spontaneously develop into their CRPC forms. PDX tissues were collected at various time points during this developmental process and subjected to comparative transcriptomic analysis. We found that *ZRSR2* is consistently and significantly upregulated in our PDX models during CRPC development and is also elevated in multiple clinical CRPC cohorts. *ZRSR2* is short for zinc finger CCCH-type, RNA binding motif and serine/arginine rich 2 and encodes a splice factor involved in the recognition of 3’-intron splice sites. *ZRSR2* has not been previously reported as involved in solid cancers but sees frequent mutations in hematologic malignancies [6]. Here, we provide evidence that *ZRSR2* could be an early driver of CRPC development.

## Materials and methods

### PDX models and microarray data

This study followed the ethical guidelines stated in the Declaration of Helsinki. Specimens were obtained from patients with their informed written consent following a protocol (#H09-01628) approved by the Institutional Review Board at the University of British Columbia (UBC). Animal studies were approved by UBC’s Animal Care and Use Committee under protocol #A17-0165. All PCa PDXs used in this study were previously established in male non-obese diabetic/severe combined immunodeficient (NOD/SCID) mice (NOD.CB17-Prkdcscid/J) and stored in liquid nitrogen as frozen seeds. Gene expression analysis was performed by microarray using the GE 8x60K Microarray as previously described [5].

Primers for qRT-PCR used in this study are listed in Supplementary Table 1. Detailed information for other materials and methods is provided in Supplementary Information.

## Results

### *ZRSR2* overexpression is an early and consistent event during CRPC development in PDXs

**Fig. 1:**
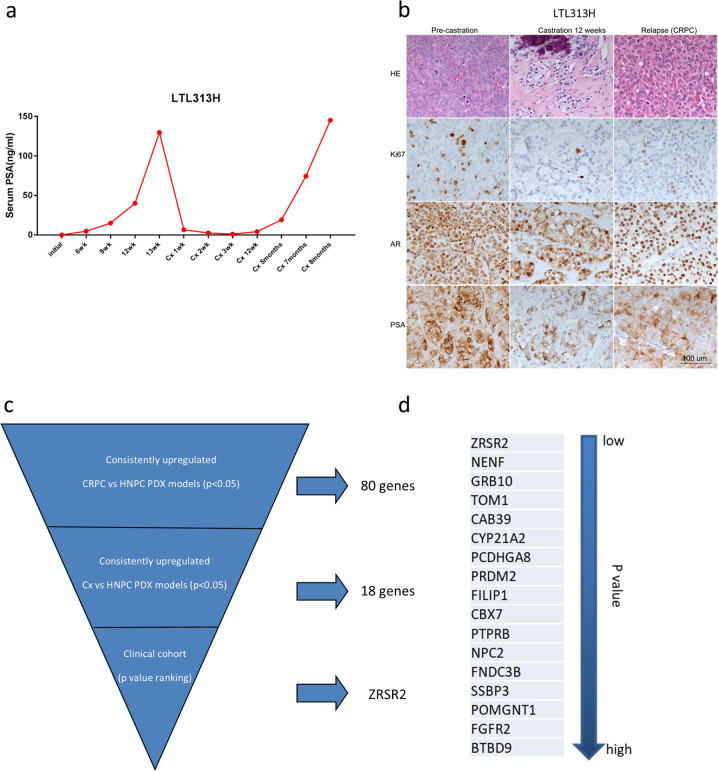
Identification of *ZRSR2* overexpression as a potential early and consistent event during CRPC development. **a** Mouse serum PSA levels at various time points before, during, and after CRPC development in the LTL-313H model. **b** HE staining of LTL-313H tumor sections with immunohistochemistry showing the expression levels of Ki67, AR, and PSA in tumor sections at various time points. **c**, **d** The strategy used to identify *ZRSR2* overexpression as a potential early and consistent event. Briefly, an 18-gene gene set was sorted for consistent upregulation in our CRPC PDX models compared to HNPC and at 12 weeks post-castration. Then, the *p* values of these genes were determined in a clinical cohort comparing CRPC and HNPC samples. *ZRSR2* was identified as the most significantly upregulated gene (lowest *p* value) in the CRPC clinical cohort.

We have previously developed a series of PDX models derived from patient HNPC tissues. These PDXs show high fidelity to the original patient tumors with respect to morphologies, genomics, gene expression levels, and treatment responses. They also mimic the clinical process of CRPC development [5]. For example, in the LTL-313H model, host castration resulted in a marked inhibition of AR signaling as indicated by a decrease in serum PSA levels and PSA protein expression. The LTL-313HR CRPC PDX develops ~5 months after host castration (Fig. 1a, b). Since tumor samples can be collected at different time points throughout this process, these PDX models allow us to carry out longitudinal analyses of CRPC development and offer a unique tool for tracing early molecular changes.

Samples of the different PDX models were collected at the initial HNPC stage, 12 weeks after castration (Cx), and when CRPC fully develops. Microarray analysis was performed to explore potential gene candidates driving CRPC development. Our previous study found a set of 18 genes that are significantly and consistently upregulated in 10 paired Cx vs. HNPC and 7 paired CRPC vs. HNPC PDX models [7]. Here, we assessed the expression levels of these 18 genes in the Cambridge 2015 clinical cohort. *ZRSR2* was the top-ranked gene that showed significant differential expression between clinical HNPC and CRPC based on *p* values (Fig. 1c, d). Thus, this data demonstrates that *ZRSR2* is a significantly upregulated gene before CRPC fully develops and its expression levels remain consistently elevated in CRPC tumors.

### Elevated *ZRSR2* expression is observed in multiple CRPC PDX models and patient samples

**Fig. 2:**
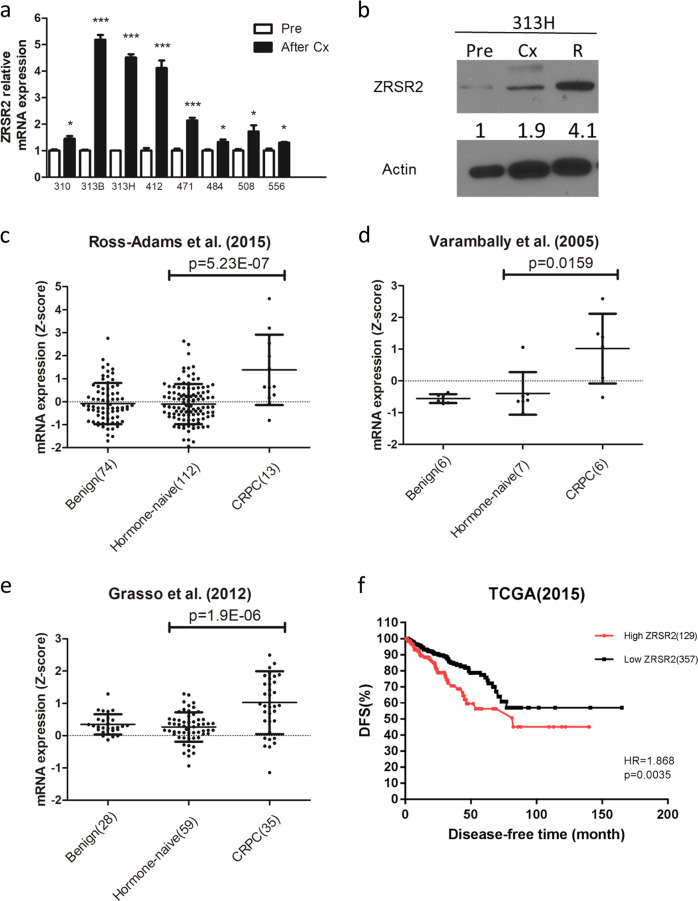
Validation of *ZRSR2* overexpression in PDX and patient CRPC tissues. **a** qRT-PCR was used to determine the expression of *ZRSR2* in multiple PDX lines after host castration. The results are presented as means ± SEM. *p* values for each sample were determined based on paired parental tumors (*p < 0.05; **p < 0.01; ***p < 0.001). **b** *ZRSR2* protein expressions in LTL-313H tumor tissues at HNPC, 12 weeks after castration, and after CRPC development were determined by western blotting. **c**, **d**, **e** *ZRSR2* mRNA expressions in benign prostate tissue, HNPC tissue, and CRPC tissue from three independent clinical cohorts. The vertical scatter plots show means ± SD. **f** Kaplan–Meier plots indicating disease-free survival times of PCa patients grouped according to *ZRSR2* expression. Significance between the groups was analyzed by the log-rank test.

*ZRSR2* mRNA expression is upregulated in multiple castrated PDX models when compared to the parental HNPC PDXs (Fig. 2a). Similarly, ZRSR2 protein expression is significantly increased during CRPC development in LTL-313H (Fig. 2b). By analyzing several publicly available clinical datasets [8], *ZRSR2* is also found to be elevated in clinical CRPC compared to HNPC in multiple cohorts (Michigan 2005, Michigan 2012, and Cambridge) [9–11] (Fig. 2c–e). Meanwhile, patients exhibiting elevated *ZRSR2* expression show shorter progression-free survival times [12] (Fig. 2f).

### Androgen deprivation therapy induces *ZRSR2* expression in PCa cells

**Fig. 3:**
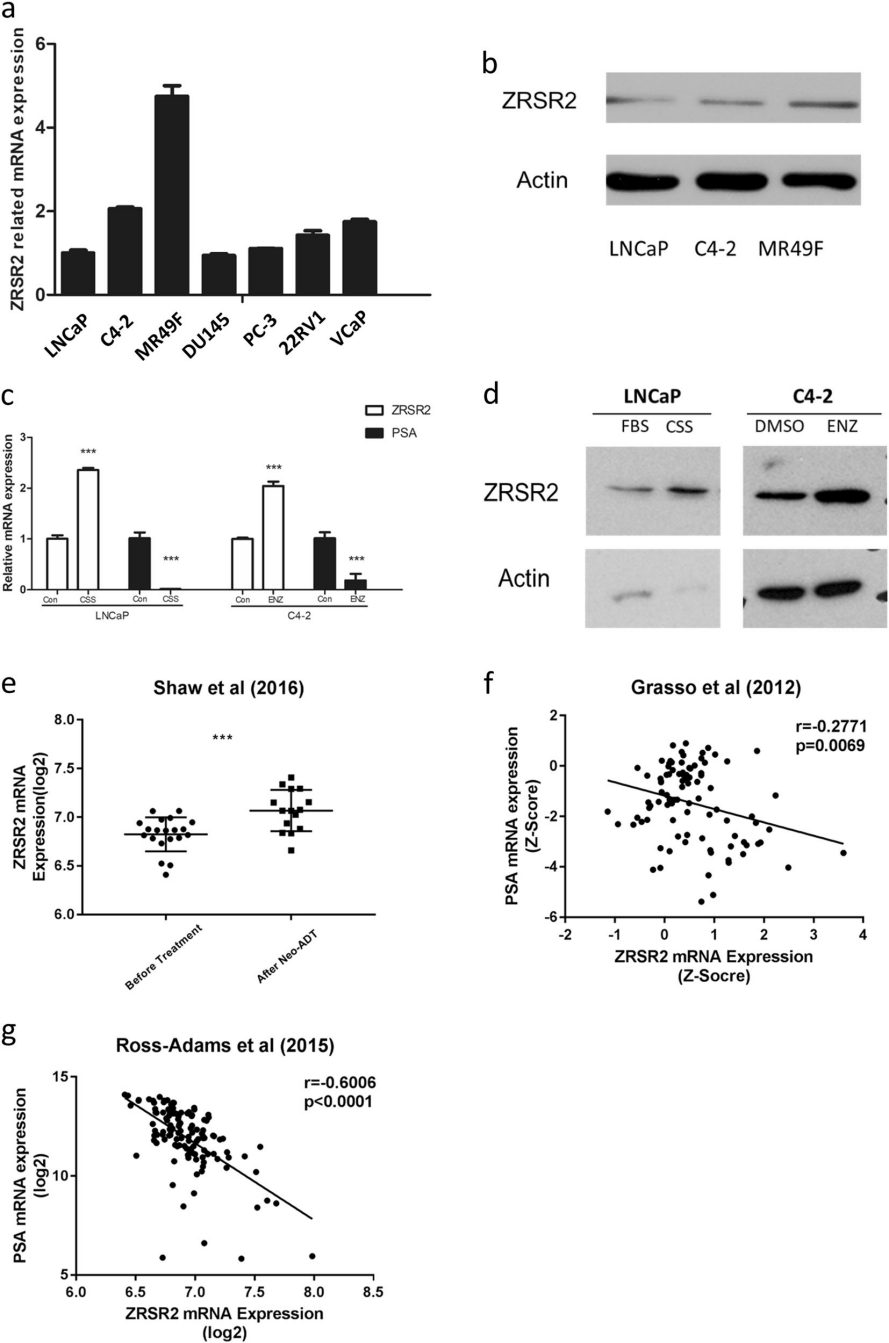
Androgen deprivation therapy induces *ZRSR2* expression in PCa cells. **a***ZRSR2* mRNA expressions in multiple PCa cell lines were determined by qRT-PCR. The results are presented as means ± SEM. **b** ZRSR2 protein expressions in the human lineage-related AR + PCa LNCaP, C4-2 and MR49F cells were determined by western blotting. **c** *ZRSR2* mRNA expressions in LNCaP cells after androgen deprivation treatment for 5 days (cultured in CSS medium) and in C4-2 cells after ENZ treatment for 5 days were determined by qRT-PCR. The results are presented as means ± SEM. **d** *ZRSR2* protein expressions in LNCaP cells after androgen deprivation therapy for 5 days (cultured in CSS medium) and in C4-2 cells after ENZ treatment for 5 days were determined by western blotting. **e** The early effect of neoadjuvant ADT (Neo-ADT) on *ZRSR2* mRNA expression in clinical PCa. The vertical scatter plots show means ± SD. **f**, **g** Pearson correlation between *ZRSR2* and *KLK3* (PSA) mRNA expressions was determined using data from public clinical cohorts. The trend line is shown.

We first determined the level of *ZRSR2* expression in multiple PCa cell lines, including the lineage-related AR + LNCaP, C4-2, and MR49F cells. We found that *ZRSR2* expression is low in the androgen-dependent LNCaP cells, higher in androgen-independent C4-2 cells, and highest in the ENZ-resistant MR49F cells at both the mRNA and protein levels. This suggests that *ZRSR2* expression in these cell lines is inversely associated with androgen independence (Fig. 3a, b). In order to examine whether *ZRSR2* expression is functionally related to androgen activity, we performed androgen ablation in LNCaP cells and androgen receptor inhibition in C4-2 cells. In androgen-sensitive LNCaP cells, *ZRSR2* mRNA and protein expressions were elevated after androgen ablation. In androgen-independent but AR signaling-active C4-2 cells, ENZ treatment also led to an upregulation of *ZRSR2* expression (Fig. 3c, d). Additionally, we found that *ZRSR2* expression is significantly upregulated in patient samples collected after neoadjuvant ADT when compared to HNPC samples [13] (Fig. 3e). Furthermore, the expression of *ZRSR2* is significantly negatively correlated with PSA expression in two independent clinical cohorts [10, 11] (Fig. 3f, g).

### Knockdown of *ZRSR2* inhibits the proliferation of PCa cells

**Fig. 4:**
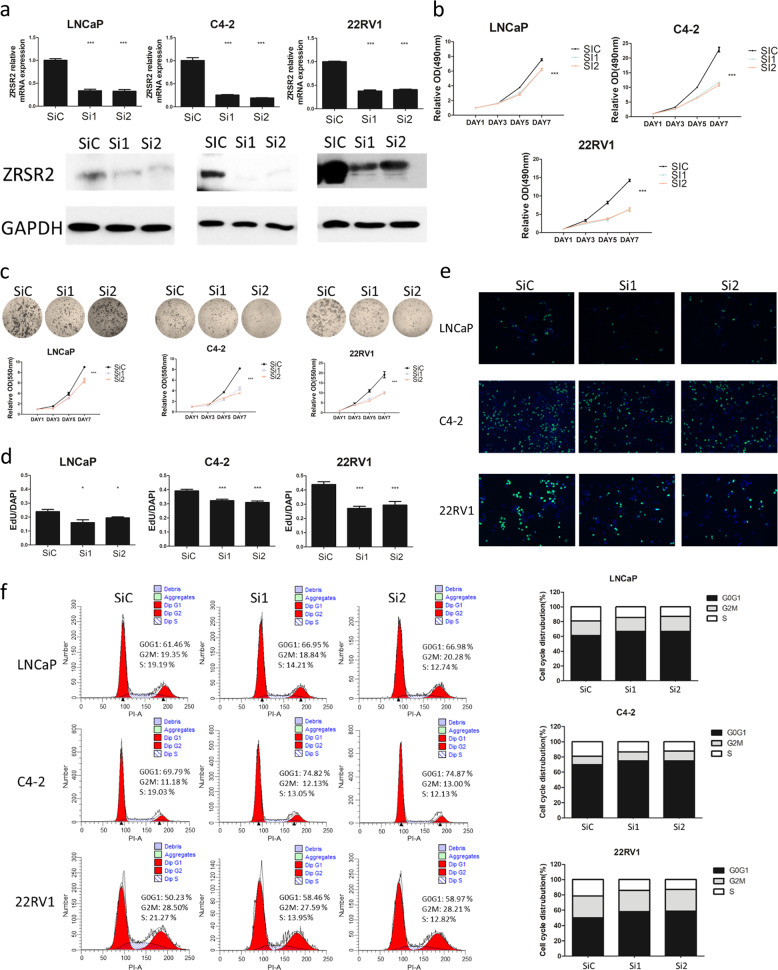
*ZRSR2* knockdown inhibits the proliferation, DNA synthesis and delays cell cycle progression of PCa cells. **a** The ability of two siRNAs to knockdown *ZRSR2* was confirmed by qRT-PCR and western blotting. The qRT-PCR results are presented as means ± SEM. **b** The proliferative effects of *ZRSR2* expression in LNCaP, C4-2, and 22Rv1 cells were determined by the MTS cell proliferation assay. OD values at 490 nm were normalized based on Day 1 readings. Results are presented as means ± SEM. **c** The proliferative effects of *ZRSR2* expression in LNCaP, C4-2, and 22Rv1 cells were further determined by the crystal violet staining assay. OD values at 550 nm were normalized based on Day 1 readings. Results are presented as means ± SEM. **d**, **e** The effects of *ZRSR2* knockdown on DNA synthesis were determined by the EdU assay. EdU-labeled cells (green) and total cells (counterstained with DAPI; blue) were counted with 10 images taken at 10-fold magnification. Bars (EdU positive/total cells) show means ± SEM. **f** FACS analysis of LNCaP, C4-2, and 22Rv1 cells after *ZRSR2* knockdown. Quantitation of cell cycle is shown in the bar graph.

Following the observation that *ZRSR2* is upregulated in CRPC, we subsequently utilized LNCaP, C4-2, and ENZ-resistant 22Rv1 cells to determine its functional role. Two siRNAs were used to knockdown *ZRSR2*. The qPCR and western blot results indicate that both sequences can successfully knockdown *ZRSR2* expression in these cells (Fig. 4a). MTS proliferation assay showed that *ZRSR2* silencing inhibited cell proliferation (Fig. 4b). Similar results were also observed using the crystal violet staining assay (Fig. 4c). *ZRSR2* knockdown inhibited cell proliferation more dramatically in androgen-independent C4-2 and 22Rv1 cells than in androgen-sensitive LNCaP cells.

### Knockdown of *ZRSR2* inhibits DNA synthesis and delays cell cycle progression in PCa cells

Knockdown of *ZRSR2* decreased DNA synthesis as indicated by a reduction in EdU-positive cells, suggesting a delayed cell cycle progression (Fig. 4d, e). Moreover, PI staining indicated that there is an increase of cells in the G0/ G1 phase and a decrease of cells in the S phase following *ZRSR2* knockdown (Fig. 4f).

### *ZRSR2* knockdown decreases *CCND1* expression in PCa cells

**Fig. 5:**
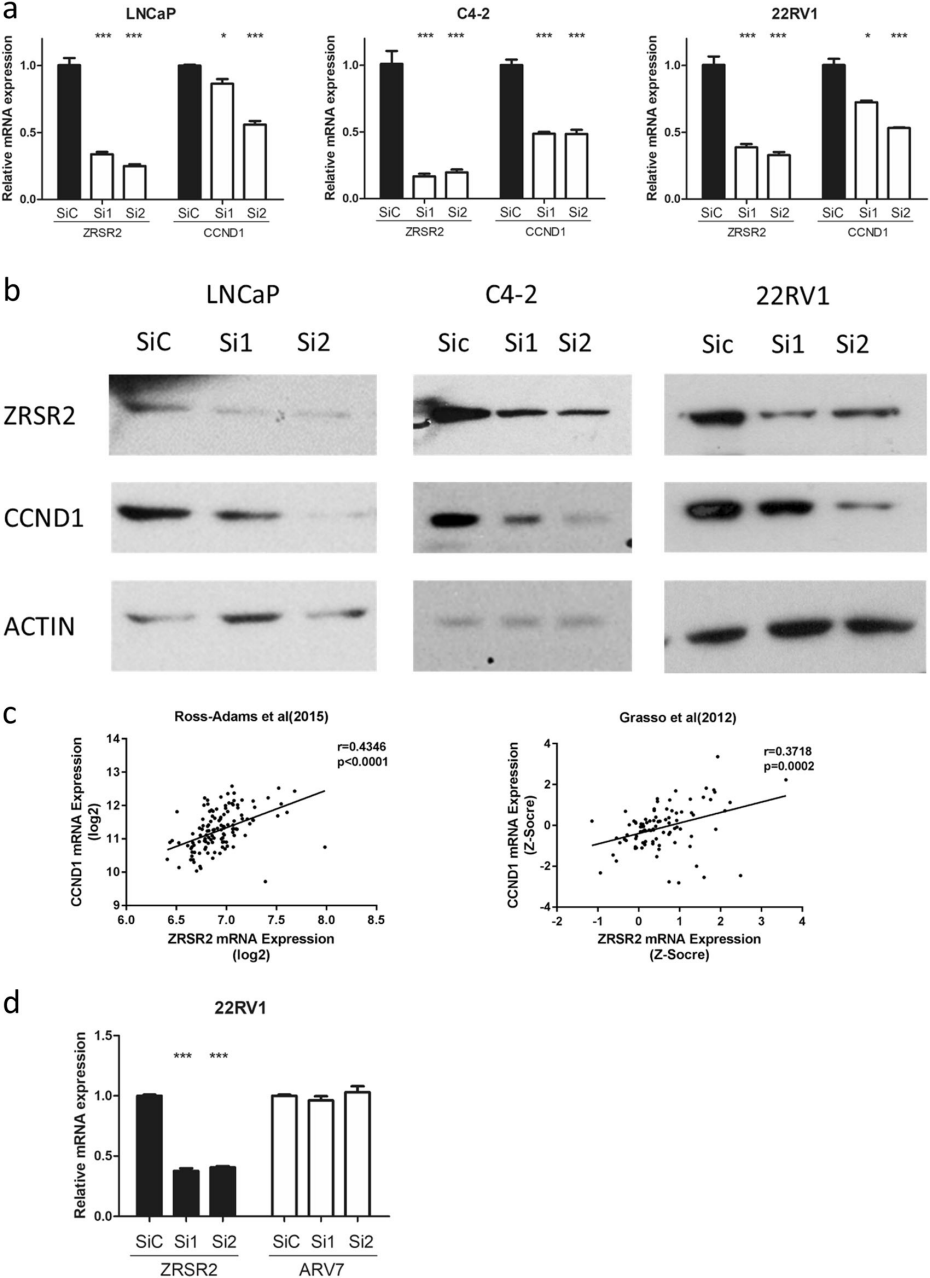
*ZRSR2* knockdown affects *CCND1* expression in PCa cells. **a** The mRNA expressions of *CCND1* after *ZRSR2* knockdown in LNCaP, C4-2, and 22Rv1 cells were determined by qRT-PCR. The results are presented as means ± SEM. **b** The effects of *CCND1* protein expression after *ZRSR2* knockdown in LNCaP, C4-2, and 22Rv1 cells were determined by western blotting. **c** Pearson correlation between *ZRSR2* and *CCND1* mRNA expressions was determined using data from public clinical cohorts. The trend line is shown. **d** The effects of AR-V7 mRNA expression after *ZRSR2* knockdown in the 22Rv1 cells were determined by qRT-PCR. The results are presented as means ± SEM.

Our data show that *ZRSR2* can affect G1 to S phase progression in the cell cycle. D-type cyclins are regulators of G1–S progression in mammalian cells [14]. Knockdown of *ZRSR2*’s partner *SF3b1* can decrease the levels of Cyclin D1 (*CCND1)* by binding U2 small nuclear ribonucleoproteins (U2 snRNP) to Tat-SF1 [15]. *CCND1* is a well-known proto-oncogene in many cancers. The *CCND1*-related signaling pathway is also significantly upregulated in CRPC [16]. Based on studies that investigated *ZRSR2* structure and binding functions, we hypothesize that *ZRSR2*’s effect on cell cycle progression in PCa may be mediated by *CCND1*. Here, we observed that knockdown of *ZRSR2* in LNCaP, C4-2, and 22Rv1 cells consistently led to a decrease in *CCND1* mRNA and protein expression (Fig. 5a, b). Furthermore, in clinical PCa samples, *ZRSR2* expression is positively correlated with *CCND1* expression in both the Cambridge and Grasso clinical cohorts (Fig. 5c). As a splicing factor, ZRSR2 knockdown did not show affect AR-V7 expression (Fig. 5d). This data suggest that upregulated expression of *ZRSR2* in CRPC might enhance *CCND1* expression, which in turn promotes cell proliferation.

## Discussion

The development of CRPC is currently a major hurdle in the management of advanced PCa [4]. A better understanding of the molecular mechanisms underlying the development of CRPC is thus needed. However, the lack of clinically relevant models, especially models based on patient-derived HNPC, is a challenge to PCa research. This is partially due to the low success rate of PCa tissue xenograft model development. Most engraftments only succeed when advanced cancers with high growth rates are used [17, 18]. To better understand the progression of CRPC, our laboratory has established 49 transplantable PCa PDX models, including models of HNPC, neuroendocrine (NE) PCa, and CRPC. We achieved this by directly grafting the patient’s tumor under the mouse renal capsule. Importantly, these PDX models show high fidelity to the original patient tumor in morphology, genomics, gene expression, and treatment response. In particular, they can mimic the progression of HNPC to CRPC upon host castration. With these PDX models, we were able to identify the common molecular events that occur during CRPC development [5, 7]. Understanding early events in tumor development can better guide disease management and control. However, these early events are difficult to identify due to the long latency in tumor development and the various limitations of PCa modeling. Our PDXs offer an excellent platform to overcome such issues. Tumor tissues from different time points following treatment can be collected for further study. Using our PDX models, we were able to identify early drivers that promoted NE transdifferentiation in prostate adenocarcinoma [19]. In this paper, using paired PDX models combined with clinical cohorts, we discovered *ZRSR2* as a potential early driver gene in CRPC development.

It is well-accepted that AR plays a major role in the progression of a majority of CRPCs [4]. In this study, we identified that *ZRSR2* expression is induced during ADT or ARPI treatment and remains elevated in AR + CRPCs. In addition, our study shows that *ZRSR2* is critical for AR + PCa cell proliferation. As shown in Fig. 4b, c, knocking down *ZRSR2* in C4-2 and 22Rv1 CRPC cells is more effective at reducing cell proliferation than in LNCaP cells. This suggests that *ZRSR2* may be essential to CRPC cells. To check the AR activity after ZRSR2 knockdown, we examined the AR target gene expression. However, there is no significant alterations with the AR target gene KLK2, KLK3, TMPRSS2 and FKBP5 after ZRSR2 knockdown (Supplementary Fig. 1). It suggested that ZRSR2-induced proliferation can be independent of AR/AR activity in CRPC.

*ZRSR2* is frequently mutated in myeloid malignancies and in ~5% of patients with myelodysplastic syndromes (MDS). However, *ZRSR2* mutations have also been found at a lower frequency in a variety of non-solid tumors [20, 21]. This study reports, for the first time, a potentially important functional role for *ZRSR2* in solid tumors. Using our unique panel of HNPC PDX models, *ZRSR2* was not only found to be upregulated in fully developed CRPC PDXs, its upregulation is also an early event after host castration during CRPC development. *ZRSR2* is rarely mutated in PCa according to public datasets in cBioPortal. Madan et al. reported that the downregulation of *ZRSR2* impaired in vitro clonogenic ability and suppressed tumor formation in mice. *ZRSR2* knockdown in erythroleukemia TF-1 and K562 cells showed a general reduction in cell growth, with fewer cells detected in the S phase of the cell cycle [6]. *ZRSR2* knockdown in HeLa cells also reduced cell viability [22]. Similarly, in our study, knockdown of *ZRSR2* in PCa cell lines inhibited cell proliferation, with an increase of cells in the G0/ G1 phase and a decrease of cells in S phase.

*ZRSR2* is an essential splicing factor involved in the recognition of 3’-intron splice sites [22]. One known mechanism of ARPI-resistance is alternative AR splicing, with AR-V7 being the most common alternative splicing product [23]. Although *ZRSR2* knockdown effectively reduced cell growth in the AR-V7 highly-expressed 22Rv1 cells, AR-V7 expression was itself not affected (Fig. 5d). ZRSR2 is a serine/arginine-rich (SR-rich) splicing factor and interacts with other SR proteins to perform its essential functions in RNA splicing [6]. Although there are no reports concerning *ZRSR2* functions in solid tumors, an increasing number of studies have reported that SR proteins and their specific kinases (SRPKs) as partners to ZRSR2-mediated splicing can have oncogenic effects in several malignancies such as lung cancer, breast cancer and PCa [24–27]. There are currently no known mechanisms regarding how *ZRSR2* mediates CRPC development. Based on our study, *ZRSR2* can facilitate cell proliferation in PCa cell lines and promote G1-to-S phase progression. Some studies reported that *ZRSR2*’s partner SRSF2 can affect *CCND1* splicing in PCa, while another partner SRPK2 can trigger cell cycle progression in neurons and induce apoptosis through regulation of nuclear *CCND1* [27, 28]. As mentioned in the results section, knockdown of SF3b1 can decrease the levels of CCND1 by binding U2 snRNP to Tat-SF1 [15]. *ZRSR2* also interacts with *SF3B1* bound to U2 snRNP [21]. A recent report demonstrated that the *CCND1* signaling pathway is significantly upregulated in drug-resistant PCa cells [16]. Here, we hypothesize that *ZRSR2* may affect cell proliferation and cell cycle progression by mediating *CCND1* expression during CRPC development. This is supported by our findings of a positive correlation between *ZRSR2* and *CCND1* expression in clinical cohorts, and a decreased expression of *CCND1* induced by *ZRSR2* knockdown.

In summary, ZRSR2 showed early and consistently overexpressed during CRPC development. ADT/ARPI treatment could induce ZRSR2 expression. As we observed, ZRSR2 was mainly associated with PCa proliferation. We hypothesized that ZRSR2 might be a promoter that is upregulated when ADT/ARPI treatment is assigned. It could protect cell proliferation and antagonize the effect of drugs then promote CRPC development. As we mentioned, most of the anti-CRPC therapies were focusing on late stage of the disease. Since the increased ZRSR2 expression was observed in early stage of CRPC development in our PDXs and clinical cohorts. It suggests an opportunity to develop a therapeutic agent that could delay or halt the development of CRPC by targeting ZRSR2 gene in combination of ADT treatment.

## Conclusion

Using PDX models, we identified, for the first time, that *ZRSR2* overexpression is an early event in CRPC development. Elevated *ZRSR2* expression is associated with poor outcomes in PCa patients. It plays an important role in CRPC development and might be implicated in *CCND1* signaling in PCa.

## Supplementary information

Supplementary Information

Supllementary figure 1

Supplementary Table 1
