# Supplementary material for: ZRSR2 overexpression is a frequent and early event in castration-resistant prostate cancer development
Source: Prostate Cancer Prostatic Dis. 2021 Feb 10;24(3):775–85. doi: 10.1038/s41391-021-00322-7 (PMC8384624; doi:10.1038/s41391-021-00322-7)
Supplement: Supplementary file 1 — Supplementary Information [file 41391_2021_322_MOESM1_ESM.doc]

**Supplementary material**

**SUPPLEMENTARY INFORMATION**

**MATERIALS AND METHODS**

**SUPPLEMENTARY FIGURE LEGENDS**

**SUPPLEMENTARY REFERENCE**

**Materials and Methods**

**Histopathology and Immunohistochemistry**

Formalin-fixed, paraffin-embedded tissue sections were prepared and analyzed by histopathology and immunohistochemistry (IHC) as previously described [^1^](#_ENREF_1). IHC staining was carried out using anti-AR (Abcam, ab108341, 1:100), anti-PSA (Santa Cruz, sc-7638, 1:100), and anti-Ki67 (Thermo Fisher, RM-9106, 1:100) antibodies. Biotinylated secondary antibodies (Vector Laboratories), peroxidase-linked avidin/biotin complex reagents (Vector Laboratories), and diaminobenzidine (DAB, Sigma-Aldrich) were used for staining.

**Cell Culture**

LNCaP, C4-2, and 22Rv1 cell lines were obtained from the American Type Culture Collection (ATCC). The cells were maintained in RPMI-1640 medium (Hyclone) supplemented with 10% FBS. To perform *in vitro* androgen ablation, LNCaP cells were cultured in RPMI-1640 supplemented with 10% CSS (Gibco). For androgen receptor blockade *in vitro*, C4-2 cells were cultured in complete medium containing 10 µM enzalutamide (ENZ). In both cases, fresh medium was added every three days. ENZ-resistant MR49F PCa cells were maintained in RPMI-1640 medium with 5% FBS supplemented with 10 μM ENZ [^2^](#_ENREF_2).

**Transient *ZRSR2* Knockdown using siRNA**

SMARTpool ON-TARGETplus Human *ZRSR2* siRNA (L-006596-02-0005, Dharmacon) and Individual 18 ON-TARGETplus Human *ZRSR2* siRNA (J-006596-18-0005, Dharmacon) were used to transiently knockdown *ZRSR2* in LNCaP, C4-2 and cells. Transfections were performed using Lipofectamine RNAiMAX following the manufacturer’s protocol.

**Total RNA Isolation, Reverse Transcription and Quantitative Real Time-PCR (qRT-PCR)**

Total RNA was extracted from cultured cells or PDX tissues using the RNeasy Mini Kit (Qiagen) following the manufacturer’s protocol. One ug of total RNA was used to synthesize cDNA using the QuantiTect Reverse Transcription Kit (Qiagen). qRT-PCR was performed using the ABI Vii 7 Real-Time PCR system (Applied Biosystems) with KAPA SYBR Fast Universal Master Mix (Kapa Biosystem). Primers used in this study are listed in Supplementary Table 1. Relative gene expression was calculated using the 2-∆∆Ct method with GAPDH as an internal reference gene.

**Western Blotting**

Total protein from cultured cells or PDX tissues was isolated using RIPA lysis buffer (50 mMTris-HCl pH 7.4, 150 mM NaCl, 1% IGEPAL, 0.5% Na-deoxycholate, 0.1% SDS) supplemented with complete protease inhibitor cocktail (Roche) and PhosSTOP phosphatase inhibitor (Roche). Protein concentration was determined using the BCA protein assay (Thermo Fisher Scientific). Total protein samples were run on an 8% SDS polyacrylamide gel, transferred to a PVDF membrane with 0.45 µm pores (Millipore), and incubated with primary antibodies overnight at 4℃. The following antibodies were used: anti-*ZRSR2* (Novus Biologicals, NBP1-57307, 1:1000), anti-CCND1 (Sigma, 05-362, 1:1000), anti-Actin (Sigma, #A2066, 1:2000), and anti-GAPDH (Santa Cruz biotechnology, sc20357, 1:1000).

**MTS Cell Proliferation, Crystal Violet Staining, and EdU Assays**

For MTS assays, cells were seeded into a 96-well culture plate (2000 cells/well) one day after *ZRSR2* siRNA transfection (marked as Day 1). The MTS Cell Titre 96 AqueousOne Solution Cell Proliferation Assay (Promega) was used to determine the cell proliferation rate. Absorbance was measured at O.D. 490 nm after 2h incubation. The relative absorbance was calculated by comparing to the absorbance readings on Day 1. For crystal violet staining, cells were seeded into a 24-well culture plate (10000 cells/well) one day after transfection. After the desired experimental period, cells were fixed in 3.7% formaldehyde (prepared from Pierce™ 16% formaldehyde, Thermo) for 30 mins and dried overnight. The fixed cells were then stained with 0.1% crystal violet solution (Sigma) for 15 mins with shaking, washed with water, and dried overnight. After solubilization with 30% Acetic acid, the absorbance of the staining solution was measured at O.D. 550nm. The relative absorbance was calculated by comparing to the absorbance readings on Day 1. For the EdU assay, transfected cells were seeded into Nunc 8-well chamber slides (Thermo Fisher) (10000 cells/ well). Two days later, the cells were fixed and stained with EdU following the Click-iTEdU Alexa Fluor 488 Imaging Kit (Thermo Fisher) protocol and imaged with AxioObserver Z1 microscope (Zeiss) (10x magnification; 10 fields). EdU positive cells (green) and Hoechst stained cells (blue) were counted using ImageJ software.

**Cell Cycle Analysis**

Cells were seeded in 6-well plates for transient *ZRSR2* or control knockdown the next day. 72h after transfection, the cells were trypsinized, resuspended in medium, and washed once with PBS. The cells were then pelleted and resuspended in 300ul of PBS, and 700ul of cold 100% ethanol was added. After being kept at -20℃ for 24h, the cells in ethanol were washed twice with PBS and incubated with 25ul/ml RNase (Sigma) and 50ul/ml propidium iodide (PI, Invitrogen) at room temperature for 30min. The DNA content was measured by flow cytometry using FACSCanto II (BD) and analyzed with the ModFit LT 4.1 software.

**Clinical Relevance Analysis**

The clinical cohorts used in this study include Grasso et al. 2012 [^3^](#_ENREF_3), Varambally et al. 2015 [^4^](#_ENREF_4), Ross-Adams et al. 2015 [^5^](#_ENREF_5), TCGA 2015 [^6^](#_ENREF_6) and Shaw et al. 2016 [^7^](#_ENREF_7). Gene expression and clinical information for the TCGA cohort were obtained through cBioPortal . Gene expression data were accessed from the Gene Expression Omnibus (GEO) database.

**Statistical Analysis**

Statistical analysis was performed using the GraphPad Prism software. The statistical difference between two groups was analyzed using the Student’s t-test (2-tailed, unpaired). Linear correlation between two groups was assessed using Pearson correlation (95% confidence interval). The Kaplan-Meier method was used for estimating progression-free survival curves. Results with p-values lower than 0.05 are considered statistically significant. The level of significance is indicated by * for p<0.05, ** for p<0.01, *** for p<0.001, and **** for p<0.0001. All experiments were performed in triplicate and repeated three times with similar results.

**SUPPLEMENTARY FIGURE LEGENDS**

Supplementary Figure 1. Additional information for AR target gene expression after ZRSR2 knockdown. The mRNA expressions of AR target gene after ZRSR2 knockdown in LNCaP, C4-2, and 22Rv1 cells were determined by qRT-PCR. The results are presented as means ± SEM.

**Supplementary Reference**

1. Lin D, Wyatt AW, Xue H, Wang Y, Dong X, Haegert A et al. High fidelity patient-derived xenografts for accelerating prostate cancer discovery and drug development. Cancer research 2014; 74(4): 1272-1283.

2. Yamamoto Y, Loriot Y, Beraldi E, Zhang F, Wyatt AW, Al Nakouzi N et al. Generation 2.5 antisense oligonucleotides targeting the androgen receptor and its splice variants suppress enzalutamide-resistant prostate cancer cell growth. Clinical cancer research : an official journal of the American Association for Cancer Research 2015; 21(7): 1675-1687.

3. Grasso CS, Wu YM, Robinson DR, Cao X, Dhanasekaran SM, Khan AP et al. The mutational landscape of lethal castration-resistant prostate cancer. Nature 2012; 487(7406): 239-243.

4. Varambally S, Dhanasekaran SM, Zhou M, Barrette TR, Kumar-Sinha C, Sanda MG et al. The polycomb group protein EZH2 is involved in progression of prostate cancer. Nature 2002; 419(6907): 624-629.

5. Ross-Adams H, Lamb AD, Dunning MJ, Halim S, Lindberg J, Massie CM et al. Integration of copy number and transcriptomics provides risk stratification in prostate cancer: A discovery and validation cohort study. EBioMedicine 2015; 2(9): 1133-1144.

6. The Molecular Taxonomy of Primary Prostate Cancer. Cell 2015; 163(4): 1011-1025.

7. Shaw GL, Whitaker H, Corcoran M, Dunning MJ, Luxton H, Kay J et al. The Early Effects of Rapid Androgen Deprivation on Human Prostate Cancer. European urology 2016; 70(2): 214-218.

8. Gao J, Aksoy BA, Dogrusoz U, Dresdner G, Gross B, Sumer SO et al. Integrative analysis of complex cancer genomics and clinical profiles using the cBioPortal. Science signaling 2013; 6(269): pl1.

9. Cerami E, Gao J, Dogrusoz U, Gross BE, Sumer SO, Aksoy BA et al. The cBio cancer genomics portal: an open platform for exploring multidimensional cancer genomics data. Cancer discovery 2012; 2(5): 401-404.
