# Supplementary figures and images for: ZRSR2 overexpression is a frequent and early event in castration-resistant prostate cancer development
Source: Prostate Cancer Prostatic Dis. 2021 Feb 10;24(3):775–85. doi: 10.1038/s41391-021-00322-7 (PMC8384624; doi:10.1038/s41391-021-00322-7)

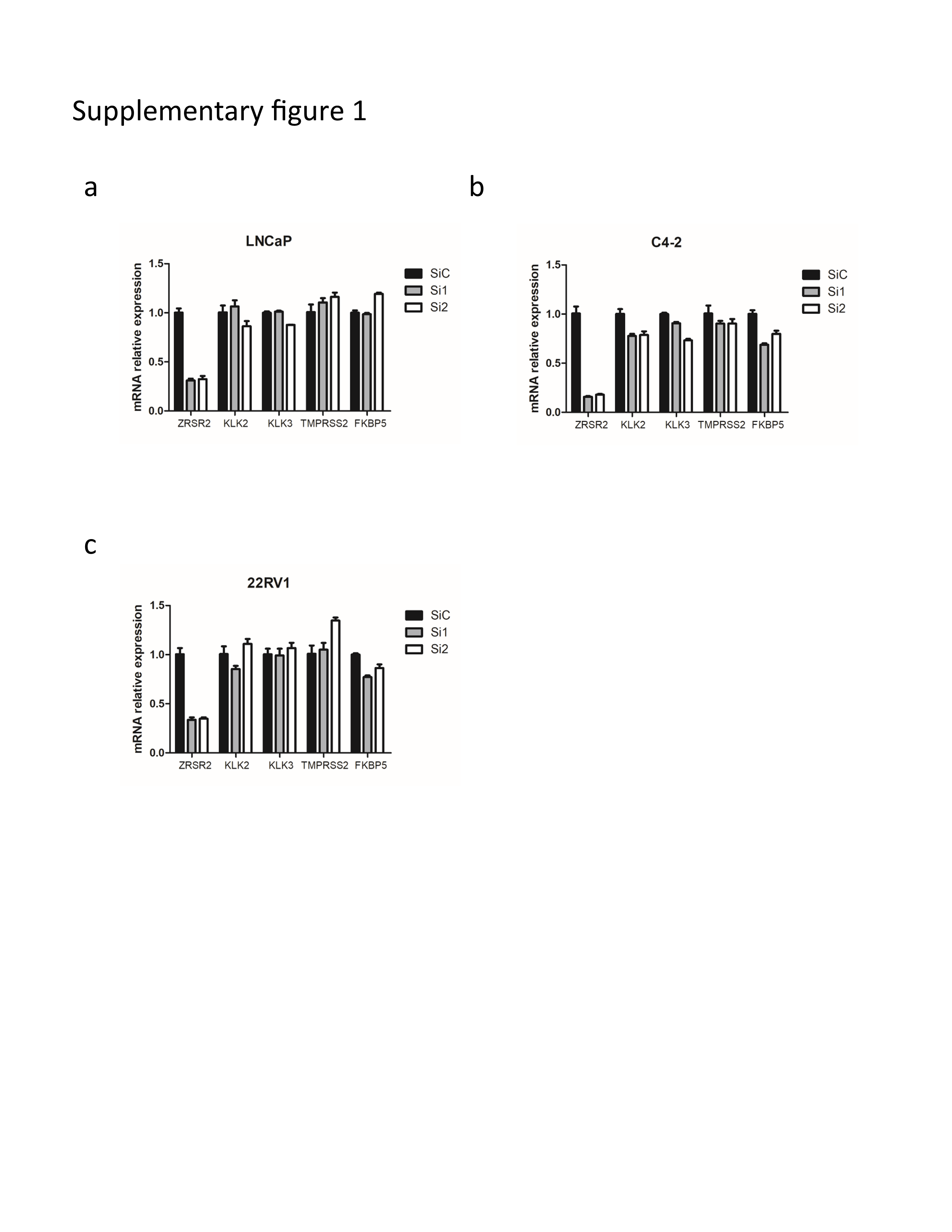

Supplement: Supplementary file 2 — Supllementary figure 1 [file 41391_2021_322_MOESM2_ESM.tif]
