# Supplementary material for: ZRSR2 overexpression is a frequent and early event in castration-resistant prostate cancer development
Source: Prostate Cancer Prostatic Dis. 2021 Feb 10;24(3):775–85. doi: 10.1038/s41391-021-00322-7 (PMC8384624; doi:10.1038/s41391-021-00322-7)
Supplement: Supplementary file 3 — Supplementary Table 1 [file 41391_2021_322_MOESM3_ESM.doc]

**Supplementary Table 1.** Primer sequences for qRT-PCR.

| Primer | Sequence |  |
| --- | --- | --- |
| ZRSR2 | Forward: 5'- CCACCACGACGACTACTACA-3' |  |
|  | Reverse: 5'- CCCCCTGTGACGACTACTT-3' |  |
| CCND1 | Forward: 5'- CCCTCGGTGTCCTACTTCAAA-3' |  |
|  | Reverse: 5'- GAAGACCTCCTCCTCGCACT-3' |  |
| PSA | Forward: 5'- CGGTTGTCTTCCTCACCCTG-3' |  |
|  | Reverse: 5'- TTGGGAATGCTTCTCGCACT-3' |  |
| AR | Forward: 5'- TCTTGTCGTCTTCGGAAATGT-3' |  |
|  | Reverse: 5'- AAGCCTCTCCTTCCTCCTGTA-3' |  |
| ARV7 | Forward: 5'- CAGGGATGACTCTGGGAGAA-3' |  |
|  | Reverse: 5'- GCCCTCTAGAGCCCTCATTT-3' |  |
| KLK2 | Forward: 5'- CCATGCCTGGAGACATATCA-3' |  |
|  | Reverse: 5'- TCCAGCACATGTCACTCTCC-3' |  |
| TMPRSS2 | Forward: 5'- GTGAAAGCGGGTGTGAGGAG-3' |  |
|  | Reverse: 5'- CTGTGCGGGATAGGGGTTTT-3' |  |
| FKBP5 | Forward: 5'- GCGGAGAGTGACGGAGTC-3' |  |
|  | Reverse: 5'- TGGGGCTTTCTTCATTGTTC-3' |  |
| GAPDH | Forward: 5'- CACCAGGGCTGCTTTTAACTC-3' |  |
|  | Reverse: 5'- GACAAGCTTCCCGTTCTCAG-3' |  |
